# Life Course Pathways of Adversities Linking Adolescent Socioeconomic Circumstances and Functional Somatic Symptoms in Mid-Adulthood: A Path Analysis Study

**DOI:** 10.1371/journal.pone.0155963

**Published:** 2016-05-23

**Authors:** Frida Jonsson, Miguel San Sebastian, Lotta M. J. Strömsten, Anne Hammarström, Per E. Gustafsson

**Affiliations:** Department of Public Health and Clinical Medicine, Unit of Epidemiology and Global Health, Umeå University, Umeå, Sweden; Leibniz Institute for Prevention Research and Epidemiology (BIPS), GERMANY

## Abstract

While research examining the health impact of early socioeconomic conditions suggests that effects may exist independently of or jointly with adult socioeconomic position, studies exploring other potential pathways are few. Following a chain of risk life course model, this prospective study seeks to examine whether pathways of occupational class as well as material and social adversities across the life course link socioeconomic disadvantage in adolescent to functional somatic symptoms in mid-adulthood. Applying path analysis, a multiple mediator model was assessed using prospective data collected during 26 years through the Northern Swedish Cohort. The sample contained 987 individuals residing in the municipality of Luleå, Sweden, who participated in questionnaire surveys at age 16, 21, 30 and 42. Socioeconomic conditions (high/low) in adolescence (age 16) were operationalized using the occupation of the parents, while occupational class in adulthood (manual/non-manual) was measured using the participant’s own occupation at age 21 and 30. The adversity measurements were constructed as separate age specific parcels at age 21 and 30. Social adversity included items pertaining to stressful life events that could potentially harm salient relationships, while material adversity was operationalized using items concerning unfavorable financial and material circumstances. Functional somatic symptoms at age 42 was a summary measure of self-reported physical symptoms, palpitation and sleeping difficulties that had occurred during the last 12 months. An association between socioeconomic conditions at age 16 and functional somatic symptoms at age 42 (*r* = 0.068) which was partially explained by people’s own occupational class at age 21 and then material as well as social adversity at age 30 was revealed. Rather than proposing a direct and independent health effect of the socioeconomic conditions of the family, the present study suggests that growing up in an unfavorable socioeconomic environment might be a source for a chain of adverse material and social living situations, which in turn affects adult health.

## Introduction

Not only a person’s current occupational class, education or income, but also the socioeconomic circumstances they encountered prior in life have been found to be important for self-rated health in adulthood [1–3]. While this research suggests that early socioeconomic conditions might affect later health independently of or jointly with adult socioeconomic position, studies examining other potential pathways are few. In accordance with a social chain of risk life course model, hypothesizing that adulthood health might be affected by a continuity of unfavorable life circumstances [4], this prospective study seeks examine if pathways of occupational class as well as material and social adversities across the life course linked socioeconomic disadvantage in adolescence to functional somatic symptoms in mid-adulthood.

The life course framework emphasizes the long-term effects of early exposures, and highlights for example the importance of disadvantage during specific life course periods and chains of unfavorable conditions across life in relation to later health [4]. Following a *sensitive* (or *critical) period* life course model, which hypothesize on enduring bodily damage and irreversible effects on adulthood health, a large body of research has examined whether socioeconomic conditions early in life may affect adult health independently of later exposures. Whether examined through parents’ education, income, wealth, some occupational based class measures or an index [5, 6], studies suggests that the socioeconomic conditions of the family during childhood/adolescence may be particularly influential for a variety of health outcomes in adulthood [1–3, 7–17]. Besides the idea of a sensitive period, however, the impact of early socioeconomic conditions might also ripple across the life course due to a continuity of unfavorable life circumstances. Consistent with the *social chain of risk* (or *pathway*) life course model, health may be a result of pathways which unfold across life due to the fact that one hardship often leads to another [4], indicating that adverse life circumstances are temporally related and persists across the life span. While an extensive amount of research has investigated if the socioeconomic conditions of the family has direct and independent effects on adulthood health, only a few studies have examined whether they might be the foundation for a series of unfavorable life circumstances. Tsenkova, Pudrovska, & Karlamangla [18] suggest that early socioeconomic disadvantage predict physical inactivity and depressive symptoms, which is in turn can lead to diabetes type 2 later in adulthood. Hagger-Johnson and colleagues [19] propose that the paternal class predicts people’s own occupational class, which in turn is linked to later health behaviour and BMI, ultimately affecting inflammatory markers later in life. Moreover, adolescents growing up in less affluent homes seem to experience more social and material stressors across the life course which has been linked to metabolic syndrome in adult women [20]. By examining the independent effects of various determinants across life in relation to poorer self-rated health [21] and with regard the risk of heart attack in later life [22], additional pathways have been proposed. Studies of this kind do not consider the temporal relation among the exposures, however, and consequently they provide limited insight to whether such pathways may constitute a potential chain of risk.

The health impact of socioeconomic circumstances is often considered to be the result of a long-term exposure to unmanageable and sustained stress [23, 24]. But since stress is a general mechanism not restricted to a certain disease or illness [25], the life course impact of socioeconomic conditions during childhood/adolescence has been linked to a variety of health problems, albeit rarely with respect to functional somatic symptoms. Functional somatic symptoms is generally described as a clustering of physical complaints without a confirmed pathological origin [26]. It has been positively associated with increased levels of stress [27] and effects a person’s health status as well as quality of life [28]. Previous studies have found it relevant to examine stress-related health problems such as psychological health [29], allostatic load [30] and even functional somatic symptoms [31] through a life course perspective. Thus, functional somatic symptoms may be a health outcome that is relevant to study as a consequence of adversity across the life course.

Following the chain of risk life course model, and by using path analysis on prospective longitudinal data, the aim of the present study is to examine whether pathways of occupational class as well as material and social adversities across the life course connects socioeconomic disadvantage in adolescent with functional somatic symptoms in mid-adulthood.

### Conceptual framework

**Fig 1:**
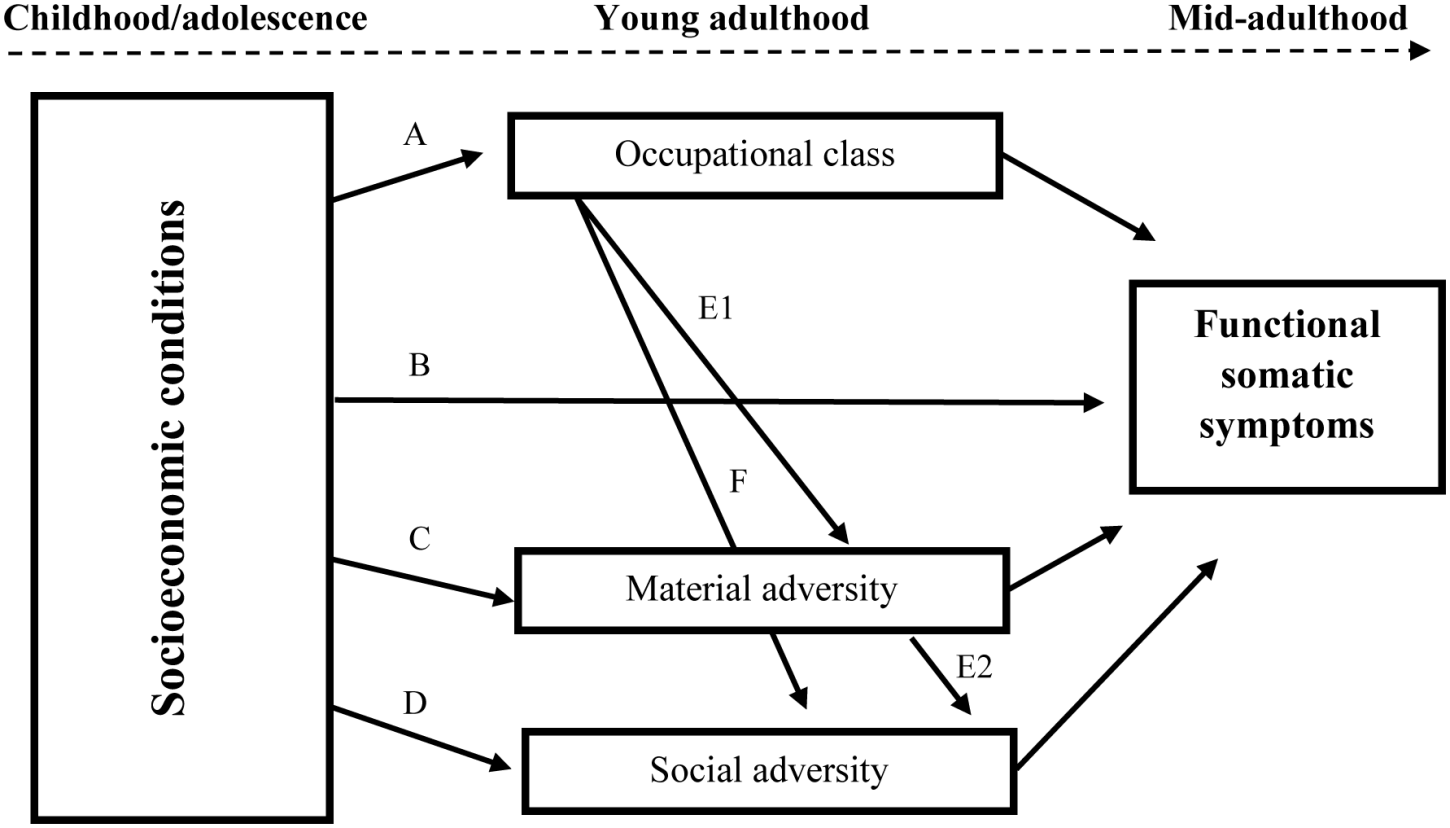
Conceptual model of adversity pathways in the association between socioeconomic circumstances early in life and functional somatic symptoms in adulthood.

Based on a large body of research suggesting that the socioeconomic conditions of the family is important for later health, either independently or by being the source of a series of unfavorable life circumstances, we developed a conceptual framework in which several plausible chains of risks are examined simultaneously (Fig 1).

First (path A) we address the likelihood that people’s socioeconomic background, as indicated by their parents occupational class, has an impact on the positions in the social hierarchy they will occupy along their life course. Although some differences exist between nations and across time, research suggests that about 12–21 percent of variation in the son’s occupational class can be attributed to the class of the father [32]. To follow the sensitive period life course hypothesis, we also included path B to capture the possibility that an adverse socioeconomic background might predict functional somatic symptoms years later, irrespective of other circumstances happening along the life course.

The concept of class proposes that occupations can be hierarchically categorized based on differences in employment relations and conditions within the labour market [33]. As indicated by the social gradient, the health of the population differs between each vertical step along the occupational structure [34]. People higher up in the social hierarchy tend to be healthier and live longer, while a disproportionately large burden of disease is concentrated amongst the most socially disadvantaged groups.

Occupational class is one of the most prominent determinants of health, and by influencing an array of material [35] and social aspects [36] of everyday life, it is often viewed as a factor distributing such resources [37]. Although this chain of risk is well established, theoretically, few have examined it empirically. Through paths E1 and F, rather than suggesting that low occupational class affects health directly (as in path A and B), we hypothesize that it predicts material and social adversities along the life course. Within the present study, material adversities are viewed as circumstances including a possession of, or an access to financial and economic resources [38], while social adversities concern stressful relational incidents or conditions that may alter a person’s usual activities and impair important relationships [39]. In light of studies suggesting that people who are exposed to such adverse life events tend to experience more stress-related health problems [40], we propose that these circumstances might be what connects class with functional somatic symptoms (paths E1 and F).

In addition to people’s own occupational class in adulthood determining aspects of their social and material lives, the socioeconomic conditions of their family during adolescence might also be a source for such adversities. Research shows that people from unfavorable socioeconomic backgrounds tend to experience more external material and interpersonal stressors [41]. Following this knowledge, and in line with previous studies [35], in path C we hypothesize that early socioeconomic disadvantage increases the risk of, for example, unemployment and financial hardship. Circumstances that may in turn predict higher levels of functional somatic symptoms in adulthood. Path D is based on a similar line of reasoning, but here we propose that growing up under unfavorable socioeconomic circumstances may also bring about social stressors such as, for example, isolation and a limited ability to have influence over one’s life [42]. In path E2, we follow previous research suggesting that social stressors might be triggered more directly by a lack of material resources [43]. Thus, the model also hypothesizes that early socioeconomic disadvantage might contribute to materially strained living conditions, which then contributes to an adverse social environment, ultimately affecting adulthood health.

## Methods

The analysis was based on prospective self-administrated questionnaire data collected during 26 years through the Northern Swedish Cohort [44]. The cohort was initiated in 1981 and included all ninth grade students (aged 16 at the time) attending compulsory school in the municipality of Luleå, Sweden. Follow-up surveys were carried out in 1983, 1986, 1995 and 2007, and out of the initial 1071 students, 94.3% participated across the entire period (n = 1010). The Northern Swedish Cohort has been granted ethical approval by the Regional Ethical Review Board in Umeå (dnr 07–057). Answering the survey was considered as consent to participate.

For the present study, data from the surveys carried out in 1981, 1986, 1995 and 2007 were used (respondents were aged 16, 21, 30 and 42, respectively) and the analysis was based on a sample of 987 individuals.

### Measures

#### Occupational class

As an indicator of the socioeconomic conditions of the family in which people grew up, the occupation of the parent’s when the cohort participant was 16 years was used as a proxy. Occupational class across mid-life was assessed using the participant’s own occupation at age 21 and 30. The coding was done in accordance with the socioeconomic classification system of Statistics Sweden [45], differentiating between workers in manual labor (blue-collar), non-manual employees (white-collar) and the self-employed. At age 16, the classification was based on an older version of the classification scheme [46]; lower class (1) was defined as having two parents working in manual labor, while having at least one parent in non-manual or self-employed occupation indicated higher class (0). At age 21 and 30, participants reporting that they were working in manual labor were classified as having low class (1) while non-manual employees and the self-employed were clustered as being of high class (0). For some participants at age 21 and 30, there was no current occupation (due to unemployment, studies or military service), and in cases where the previous occupation was not accessible, the highest educational attainment was used as a proxy (at age 21, *n* = 206 and at age 30, *n* = 41). High class (0) was indicated by university studies or a high school degree qualifying the person for university studies. Low class (1) reflected all other high school degrees or lower levels of education.

#### Life course measures of social and material adversity

The operationalizations has been previously used elsewhere [47, 48], and were based on items from age specific questionnaires completed by the participants at age 21 and 30 making the set adversities differ slightly between the measures. Parcels were created by adding up the number of adversities for each concept and age while acknowledging that, as holistic constructs, the concepts are theoretically and empirically heterogeneous.

#### Social adversity

Social adversity at age 21 included items pertaining to *residential instability* (the number of times the respondent had moved during the last 3 years) and exposure to *illness and death* during the last 3 years (defined as someone close to the respondent suffering from serious or long-term illness and/or if someone close to them had died). At age 30, social adversity included items pertaining to exposure to *illness and death* during the last 12 months; experiencing a *separation* during the last 12 months (defined as breaking up from a long-term cohabitating relationship); *social isolation* (the total score on four items in the Availability of Social Integration scale of the Interview Schedule for Social Interaction [49]); having *low decision latitude* (the summary response to six four-level Likert scale items about decision latitude, four on skill discretion and two on decision authority [50]; or being *exposed to threat/violence* during the last 12 months (due to low frequencies, defined as a positive response on either of four items was used: 1) personally being abused at work through mean words and actions from bosses or colleagues, 2) sexual harassment through unwelcome or degrading sexual insinuations, 3) physical violence or 4) threats of violence that were so serious that she or he was scared). Social adversity at age 21 ranged from 0–3 for both women and men, and at age 30 between 0–5 for men and 0–6 for women.

#### Material adversity

Material adversity was operationalized by drawing on information pertaining to: *unemployment*, at both age 21 and 30, defined as currently being unemployed or on disability pension; also, *low cash margin*, not being able to raise 5,000 SEK and 13,000 SEK within a week, at age 21 and 30, respectively. At age 30, items pertaining to *spousal unemployment*, defined by the participant’s partner being unemployed during the last 5 years, and *financial strain* was also included. The latter was captured through a question regarding how often (answered ‘often’, ‘seldom’, ‘never’, or ‘not applicable’, where the number of ‘often’ responses were dichotomized at the 80th percentile) the respondent had abstained from any of 11 different material needs due to monetary problems (for example, eat a cooked meal, buy clothes, and pay the rent or other bills due to financial reasons). After being summarized, the measures ranged between 0–3 at age 21 and 0–4 at age 30 for both sexes.

#### Functional somatic symptoms

The primary outcome, functional somatic symptoms at age 42, was a summary measure of ten different symptoms (cardiopulmonary/autonomic, gastrointestinal, musculoskeletal and general symptoms) occurring during the last 12 months. Each symptom was coded 0–2, and collected through three survey questions. For eight symptoms the following question was used: ‘Do you have (or have you during the last 12 months) had any of the following symptoms: headache or migraine; other stomachache; nausea; backache, hip pain or sciatica; fatigue; breathlessness; dizziness; overstrain. The response options were ‘No’ (0), ‘Yes, light’ (1) and ‘Yes, severe’ (2). Palpitation was covered using the question: ‘How often have you had nervous problems during the past 12 months’, with the type of symptom and frequency indicated as ‘never’ (0), ‘sometimes’ (1) and ‘always’ (2). For sleeplessness, the following question was used: ‘Have you had sleeping difficulties during the past 12 months?’ with the response options coded as ‘Never’ (0), ‘Sometimes’ (1) and ´often’ or ‘always’ (2). When summarized, the variable ranged from 0–18 for women and 0–15 for men, with higher values corresponding to more somatic problems. Cronbach’s alpha revealed an internal consistency of 0.782.

#### Control variables

Sex and functional somatic symptoms at age 16 were used as control variables. The operationalization of functional somatic symptoms was identical to the one used at age 42 (see description above), but based on questionnaires completed by the participant at age 16.

### The empirical model

**Fig 2:**
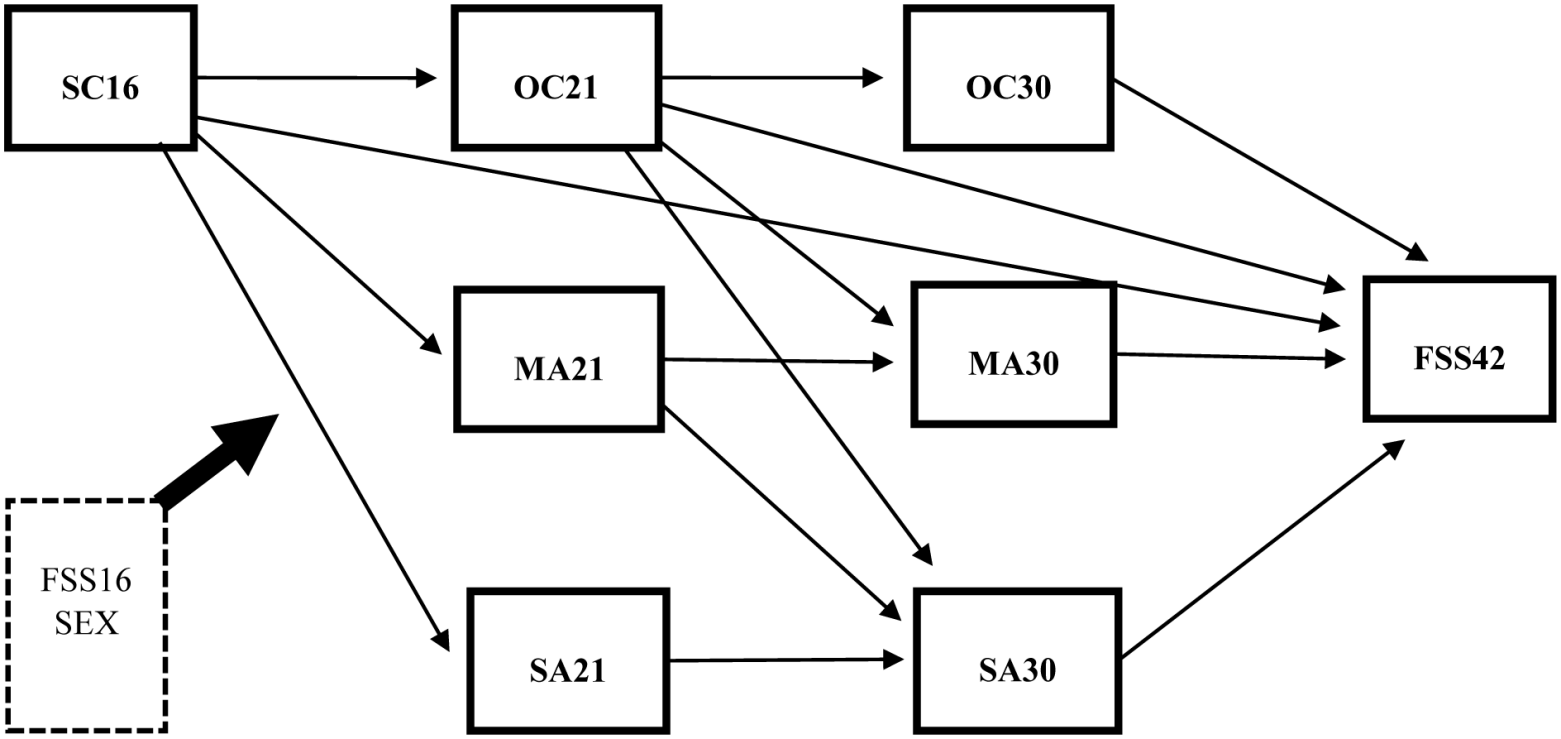
The multiple mediator model examining the relation between socioeconomic conditions at age 16 (SC16) and functional somatic symptoms at age 42 (FSS42), via occupational class (OC21 and OC30), material adversity (MA21 and MA30) and social adversity (SA21 and SA30) at age 21 and 30. Paths are estimated direct effects between the variables, while controlling for sex and the baseline functional somatic symptoms for the respondents at age 16 (FSS16) (n = 987).

As an operationalization of the conceptual framework (Fig 1), one multiple mediator model (Fig 2) was developed containing several hypotheses, which were combined and examined simultaneously [51].

First, to examine social and material adversities as plausible mediators, ten manifest variables were connected through direct paths based on their temporal precedence [52]. Three variables were independent: socioeconomic conditions at age 16, and the control variables sex and functional somatic symptoms at 16. Seven were dependent; two binary measures—occupational class at age 21 and 30 —and five continuous measures—material and social adversity at age 21 and 30 –as well as functional somatic symptoms at age 42.

Second, as personal characteristics as well as social support may be potential sources of error [53], residuals of measures at age 21 and 30 were allowed to covary [51]. Within the model, all the paths were adjusted for sex and baseline functional somatic symptoms (age 16). Since social and psychological resources may vary according to gender [54], and given that men and women tend to experience and cope with adversity slightly differently [55], the analysis was also stratified by sex.

### Data analysis

To analyze the empirical model (Fig 2), path analysis using Mplus 7 [56] was applied, enabling us to examine a multiple mediator model with a combination of binary and continuous variables while estimating all parameters simultaneously [57]. By considering categorical measures to be indicators of a latent continuous variable, dichotomized at a threshold value, binary variables could be estimated in combination with continuous variables. To attain path coefficients and model fit, WLSMV (a robust, mean- and variance-adjusted weighted least square method) was used with THETA parameterization [56].

The model-data correspondence was evaluated relative to a number of fit indices. The chi-square (x^2^) was used to examine the hypothesis of exact fit [58] while the value and confidence interval of the root mean square error of approximation (RMSEA) provided complementary information on the additional close fit hypothesis [59]. By also considering the comparative fit index (CFI) as well as the weighted root mean square residual (WRMR), our empirical model was examined against a baseline model that was based on the assumption that all the variables were uncorrelated. In accordance with Yu [60], RMSEA had to be around .06, the CFI close to .96 and a WRMR had to be smaller than 1.0 for model fit to be acceptable.

Within the model, the estimates of the direct effects are probit regression coefficients when the dependent variable is categorical, and linear regression coefficients when they are continuous. Mediation was examined using a percentile bootstrap, a nonparametric re-sampling technique that provided 95% bootstrapped confidence intervals for indirect effects and bootstrapped standard errors (5,000 samples requested) [51]. Significant effects were displayed at the *p* < 0.05 level, provided that the confidence interval did not include 0 (zero).

Selection bias regarding missing data was examined, analyzing whether the effective sample differed from respondents excluded because of incomplete data. The main variables were used as predictors of a binary missingness variable through simple logistic regression in SPSS 21. Two measures—socioeconomic position at age 30 (*n* = 21 excluded, *p* = 0.047) and social adversity at age 30 (*n* = 12 excluded, *p* = 0.030)–revealed significant estimates, indicating that the missing data is MAR, i.e. conditional only on covariates in the model. Since simulation studies have shown that weighted least square estimation using pairwise deletion provide accurate estimates under the MAR assumption [61] this method was used to handle our missing data.

Preliminary data screening revealed no severe violations to the normality (skewness < 1.1, kurtosis < 2.0) or linearity assumptions, and as indicated by a low variance inflation factor (VIF) (values < 1.1) multicollinearity was not present. Since the direction and inclusion of paths were guided by theory, a sensitivity analysis was performed to examine whether we had been too restrictive in the operationalization of the conceptual framework. Rerunning a saturated model that allowed for all plausible pathways did however not change the results of the study, why the original model was retained and reported in the results section.

## Results

Table 1 presents descriptive statistics for the variables in the model, on the full sample and stratified by sex. Respondents with origins in a high socioeconomic setting were in the majority (62%), as indicated by the occupation of their parent’s. At age 21, the reverse was apparent and more than half reported a low occupational class of their own (63%), while at age 30, this number had decreased to 43%. The sex stratified analysis (Table 1) revealed that social and material adversity as well as functional somatic symptoms at age 42 differed, where women presented more adversities overall, as well as more functional somatic symptoms at age 42, compared to men.

**Table 1:** Descriptive statistics; mean and standard deviation, in the full sample (n = 987) and stratified by sex. All the variables and control variables in the model at four points in time—respondents aged 16, 21, 30 and 42. Predictor estimates are mean (SD).

Variables	Full sample		Women		Men		Difference
	Range	Estimate	Range	Estimate	Range	Estimate	*p*-value
**Material adversity**							
Age 21	0–3	0.54 (0.682)	0–3	0.57 (0.702)	0–3	0.51 (0.663)	0.156 ^a^
Age 30	0–4	0.77 (0.947)	0–4	0.88 (1.029)	0–4	0.66 (0.852)	<0.0005 ^a^
**Social adversity**							
Age 21	0–3	0.76 (0.850)	0–3	0.91 (0.891)	0–3	0.62 (0.785)	<0.0005 ^a^
Age 30	0–6	0.97 (0.989)	0–6	1.04 (1.002)	0–5	0.91 (0.974)	0.031 ^a^
**Functional somatic symptoms**							
Age 16	0–16	3.35 (2.540)	0–16	3.71 (2.510)	0–12	3.03 (2.526)	<0.0005 ^a^
Age 42	0–18	4.24 (3.306)	0–18	4.67 (3.503)	0–15	3.75 (3.032)	<0.0005 ^a^
**Socioeconomic conditions**							
Age 16	0–1	37.7% low					
**Occupational class**							
Age 21	0–1	62.9% low					
Age 30	0–1	43.2% low					
**Sex**							
Women	1	48.2%					
Men	2	51.8%					

^a^ p-value based on t-test

Table 2 presents zero-order correlation coefficients (Pearson’s *r*) between all the variables. Cross-validation using Spearman’s rho yielded similar results. Although only marginally, an unfavorable socioeconomic environment at age 16 was positively correlated with functional somatic symptoms at age 42 (*r* = 0.068), while both material and social adversities at age 30 were significantly related to socioeconomic conditions in adolescence (*r* = 0.192 and 0.123, respectively) as well as functional somatic symptoms at age 42 (*r* = 0.191 and 0.205, respectively).

**Table 2:** Pearson’s correlations for socioeconomic conditions (SC), occupational class (OC), material adversity (MA), social adversity (SA), and functional somatic symptoms (FSS) at four points in time—respondents aged 16, 21, 30 and 42.

**Variables**	**SC16**	**OC21**	**OC30**	**MA21**	**MA30**	**SA21**	**SA30**	**FSS16**	**FSS42**
**SC16**	-	0.229**	0.205**	0.055	0.192**	0.033	0.123**	0.042	0.068*
**OC21**		-	0.426**	-0.009	0.188**	-0.002	0.141**	-0.006	0.060
**OC30**			-	0.063*	0.263**	-0.005	0.218**	-0.008	0.105**
**MA21**				-	0.231**	0.041	0.093**	0.003	0.026
**MA30**					-	0.140**	0.261**	0.079*	0.191**
**SA21**						-	0.080*	0.086**	0.094**
**SA30**							-	0.064*	0.205**
**FSS16**								-	0.230**
**FSS42**									-

* p < 0.05 (2-tailed),

** p < 0.01 (2-tailed)

**Fig 3:**
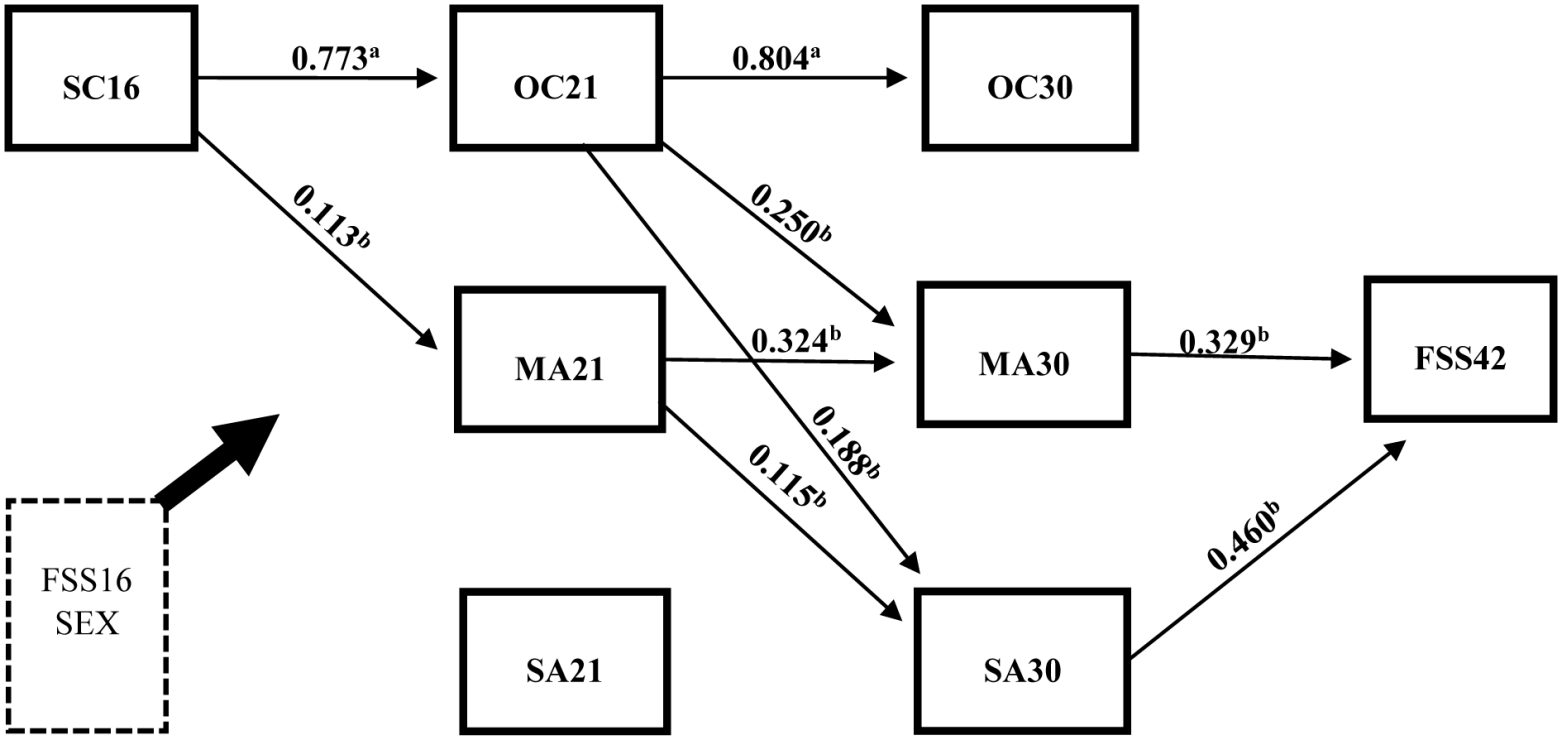
Significant (p < 0.001) path coefficients in the model estimated with respect to the full sample (n = 987) while controlling for sex and baseline functional somatic symptoms. ^a^ Predictor estimates are probit regression coefficients. ^b^ Predictor estimates are unstandardized regression coefficients (B).

The empirical model (Fig 2) was analyzed, assessing direct and indirect effects with bootstrapped confidence intervals and standard errors [62]. All fit indices, except a significant chi-square, indicated the model had a good fit (*n* = 987) to the data (x^2^ = 36.312 with *p* < 0.001 and *df* = 8, RMSEA = 0.060, CFI = 0.963 and WRMR = 0.726). Table 3 displays all the estimated direct and indirect effects, adjusted for sex and baseline functional somatic symptoms, in the full sample. In Fig 3, only significant (*p* < 0.001) path coefficients for direct effects are presented. The analysis revealed a significant association between socioeconomic conditions in adolescence and a person’s occupational class at age 21, and from age 21 to age 30, indicating a continuity of along the life course. With regard to the health impact, neither the socioeconomic conditions of the family at age 16 nor people’s occupational class at age 21 and 30 was directly related to functional somatic symptoms in adulthood. However, a person’s class at age 21 significantly predicted material as well as social adversity at age 30 (*B* = 0.250 and 0.188), which in turn related to functional somatic symptoms at age 42 (*B* = 0.329 and 0.460, respectively).

**Table 3:** Direct and indirect effects (5000 samples requested), adjusted for sex and baseline functional somatic symptoms, in the model for the full sample (n = 987). The variables are socioeconomic conditions (SC), occupational class (OC), material adversity (MA), social adversity (SA) and functional somatic symptoms (FSS), at four points in time—respondents aged 16, 21, 30 and 42.

**Total effect** ^a^	**B (S.E.)**	**CI**	**β (S.E.)**	**CI**
*SC16 → FSS42*	0.425 (0.217)	0.001, 0.850	0.063 (0.032)	0.001, 0.125
**Direct effects** ^b^	**Estimate**	**CI**		
*SC16 → FSS42*	0.179 (0.246)	-0.304, 0.662		
*SC16 → OC21*	0.773 (0.087)	0.606, 0.939		
*SC16 → MA21*	0.113 (0.044)	0.019, 0.206		
*SC16 → SA21*	0.070 (0.055)	-0.038, 0.178		
*OC21 → OC30*	0.804 (0.078)	0.650, 0.957		
*OC21 → MA30*	0.250 (0.035)	0.182, 0.319		
*OC21 → SA30*	0.188 (0.038)	0.114, 0.263		
*OC21 → FSS42*	-0.019 (0.212)	-0.434, 0.395		
*MA21 → MA30*	0.324 (0.050)	0.225, 0.422		
*MA21 → SA30*	0.115 (0.054)	0.010, 0.221		
*SA21 → SA30*	0.065 (0.037)	-0.008, 0.139		
*OC30 → FSS42*	0.178 (0.158)	-0.133, 0.489		
*MA30 → FSS42*	0.329 (0.141)	0.053, 0.606		
*SA30 → FSS42*	0.460 (0.123)	0.218, 0.701		
**Total indirect effect** ^a^	**B (S.E.)**	**CI**	**β (S.E.)**	**CI**
*SC16 → FSS42*	0.246 (0.109)	0.032, 0.461	0.036 (0.016)	0.005, 0.068
**Specific indirect effect** ^a^				
*SC16 → OC21 → OC30 → FSS42*	0.110 (0.102)	-0.089, 0.310	0.016 (0.015)	-0.013, 0.046
*SC16 → OC21 → MA30 → FSS42*	0.064 (0.030)	0.004, 0.123	0.009 (0.004)	0.001, 0.018
*SC16 → OC21 → SA30 → FSS42*	0.067 (0.024)	0.019, 0.114	0.010 (0.004)	0.003, 0.017
*SC16 → MA21 → MA30 → FSS42*	0.012 (0.008)	-0.004, 0.028	0.002 (0.001)	-0.001, 0.004
*SC16 → MA21 → SA30 → FSS42*	0.006 (0.005)	-0.004, 0.016	0.001 (0.001)	-0.001, 0.002
*SC16 → SA21 → SA30 → FSS42*	0.002 (0.003)	-0.003, 0.007	0.000 (0.000)	0.000, 0.001

^a^ Predictor estimates for the total, indirect, and specific indirect effects are unstandardized path coefficients (B) with bootstrapped standard errors (S.E.) and bootstrapped 95% confidence intervals (CI) and standardized path coefficients (β) with bootstrapped standard errors (S.E.) and bootstrapped 95% confidence intervals (CI).

^b^ Predictor estimates for the direct effects are unstandardized path coefficients (S.E.) and 95% confidence intervals (CI).

With regard to the aim of the study, plausible chains of risks were examined assessing the indirect effects. First, in the full sample, the total effect which represent the overall impact of a person’s socioeconomic conditions at age 16 on functional somatic symptoms at age 42 (irrespective of whether or not the effect runs through intervening variables) [62], was significant (*B* = 0.425, 95% CI = 0.001–0.850). The total indirect effect was also significant (*B* = 0.246, 95% CI = 0.032–0.461), suggesting a set of variables mediated the association (Table 3).

Second, by assessing the specific indirect effects, the mediating role of a person’s occupational class and their material and social adversity at age 21 and 30 in the direct association between socioeconomic conditions at age 16 and functional somatic symptoms at age 42, were obtained. The results suggested two plausible pathways from adolescent socioeconomic circumstances: 1) via occupational class at age 21 and further through material adversity at age 30 (*B* = 0.064, 95% CI = 0.004–0.123), and 2) also through class at age 21 but then via social adversity at age 30 (*B* = 0.067, 95% CI = 0.019–0.114).

Stratifying the model by sex (S1 Table) did not substantially alter the model fit (*n* = 473 and 514 for women and men respectively). Chi-square remained significant (x^2^ = 41.793 with *p* = 0.004 and *df* = 16), while all the other indices changed slightly (RMSEA = 0.057, CFI = 0.964 and WRMR = 0.829). When examined separately, the total indirect effect was significant for men (*p* = 0.008) but not for women (*p* = 0.507). Consequently, no path was evident for women, while for men an indirect pathway via occupational class at age 21 and social adversity at age 30 was significant (*B* = 0.093, 95% CI = 0.019–0.166).

## Discussion

In light of all research pointing to the importance of early socioeconomic conditions for later health, to the best of our knowledge, only a few studies [18–21] have explicitly examined how growing up in a socioeconomically disadvantaged home may affect people’s health through chains of unfavorable life circumstances. Following the chain of risk hypothesis, our study adds to the field by demonstrating that adverse socioeconomic conditions of the family may contribute to pathways of unfavorable material and social living situations, ultimately affecting health in adulthood.

The socioeconomic circumstances under which one is brought up may structure the subsequent life and affect health in many ways. Consistent with the sensitive period hypothesis, a large body of research suggests that health might be affected independently of or jointly with adult socioeconomic position [1–3, 7–17]. While our results follow these studies by proposing that the socioeconomic environment people grow up in might predict their occupational class as adults, we found no support for the idea that socioeconomic conditions of the family impact on health directly, or through a person’s own occupational class (Fig 3). Although this might be due to our fairly restrictive operationalization of early socioeconomic circumstances (parents’ occupation) or to our choice of outcome (functional somatic symptoms), it is possible that by only examining a person’s class in adulthood as a plausible pathway, previous studies have overlooked other explanatory circumstances along the life course [63]. Because as hypothesized (conceptual framework, Fig 1), this study suggests that the socioeconomic circumstances of the family can bring about a life of strained and stressful relational and financial situations, experiences that seem to stand for the more immediate effects on adult health.

The sex stratified analyses (Table 1) revealed that women reported higher levels of functional somatic symptoms, but also more adversities than men. Although this is in line with previous studies which suggest that health effects of social and material stressors may differ between the genders [54], the mechanisms are far from understood. Women’s structural disadvantage with greater exposure to social and material stressors can explain some part, while the idea women would be more susceptible and vulnerable to these stressors provide limited insight as to why stress-related health problems differ between women and men [40]. In contrast to these results, our sex stratified indirect effects suggested that by influencing the social environment in mid-adulthood, unfavorable socioeconomic circumstances early in life may increase the risk of later functional somatic symptoms, but only for men (S2 Table). Following the buffering hypothesis, notwithstanding it being fairly speculative, a reason for this finding might be that men generally have less social support and are consequently more affected by social adversity [64].

Even though poor health in adulthood may have its origins in, and be driven by the socioeconomic environment of the family during adolescence, our results suggest this is not a sole and independent determinant. Instead of proposing a permanent and irreversible damage that may only be avoided through interventions early in life, the present study highlights the possibility that by breaking the subsequent links in the chain of risk, health effects along the life course might be altered. Thus, through interventions focused on improving the social and financial living conditions for people from impoverished backgrounds, we might have a chance to avert stress-related health problems later in life.

### Methodological considerations

The over-all strengths of the study are the ability to examine a rather comprehensive theoretical model [52], assess mediation through prospective (spanning over 26 years) longitudinal data [62], a sample representativeness relative to the same age cohort in Sweden as a whole [44] and a reduced risk of selection bias due to the high response rate.

However, our study has several limitations. First, while we developed a comprehensive model and adjusted for potential confounders, not all possible variables and paths have been accounted for. We did not examine whether any measures at age 42 might have an impact on functional somatic symptoms, since this would have rendered the temporal sequence of the intermediate factors at age 21 and 30 years preceding the outcome at age 42 years less distinct. So while we tried to decrease the risk of omitted variable bias by estimating all the variables simultaneously [65] and acknowledged that they may share common omitted causes by correlating the residuals of contemporary measures [51] our estimates may still be biased as a result of variables not considered in the model [66].

Second, uncorrected measurement errors may introduce several problems in manifest path analysis [67]. Nevertheless, creating parcels enabled us to partially adjust for this potential problem, although operationalizing the adversity measures were limited to items available in the questionnaires. Thus, these variables have likely captured only a fraction of all possible hardship that may follow an unfavorable socioeconomic environment early in life. Also, by adopting a life course perspective we were required to operationalize class at age 21 which is a period of transition. Consequently, despite our class operationalization being guided by a standard classification scheme [45], the measure at age 21 is debatable since we used education as a proxy for people that did not have a current occupation. In addition, we have made a differentiation between, and assume that class is temporally prior to our adversity measures by being a source for material and social resources [35, 37]. However, developing more comprehensive measures, including other items, approaching them as latent constructs, or their interconnectedness in another way, might have yielded quite different results [52]. As methods to assess mediation are constantly evolving and improving, see for example VanderWeele [68], it is also possible that by using a causal mediation method to estimate our model (e.g. the one presented by Wang, Nelson and Albert [69]) the indirect effects might have been different.

Third, in the process of specifying the model, alternative ideas were examined—one assessing autoregressive effects and another focused on health selection. When model fit was qualitatively and subjectively compared between them, all proved to have a similarly acceptable fit. In the end, although all fit indices except the chi-square (a test that is sensitive to large sample sizes) indicated that the current model attained acceptable model-data correspondence, there are other versions that might fit the data equally well or better [70].

Lastly, growing up during adverse socioeconomic conditions was examined with regard to self-reported functional somatic symptoms in adulthood. The measure has been shown to have acceptable factorial invariance as well as internal consistency over time [71] but functional somatic symptoms is a complex concept and there is an ongoing debate about its nature, diagnosis and impact [72]. Thus, while this health problem might be representative of other somatic problems linked to stress [73], our results may not be generalizable beyond this specific outcome. In addition, functional somatic symptoms were defined as a clustering of physical complaints with no or unknown pathology. Whether the measure is actually medically unexplained is not certain, as the operationalization was based on items of self-reported symptoms, which have not been assessed in relation to the presence of an actual diagnosis. In addition, residual confounding may bias the results; for example, constitutional factors such as personality, which has been shown to relate to functional somatic symptoms [74]. However, despite these shortcomings, our measure is comparable to those commonly used in population studies [75] and, to a fairly high degree, people reporting a multiple of these problems tend to be without a medical disorder that may explain their symptoms [26].

## Conclusion

The present study expands previous literature on the health effects of early socioeconomic disadvantage by examining, and finding support for, the chain of risk life course hypothesis. Specifically, our results indicate that growing up during unfavorable socioeconomic conditions might be a source for a chain of adverse material and social living situations along the life course, and these might be the circumstances that largely explain the effects of early disadvantage on health in adulthood. Instead of proposing a permanent and irreversible impact, the present study therefore highlights the possibility that interventions focused on improving the social and financial living conditions for people from impoverished backgrounds might help avert poor health in adulthood.

## Supporting Information

S1 Table Pearson’s correlations between all the variables for women (above diagonal) and men (below diagonal); socioeconomic conditions (SC), occupational class (OC), material adversity (MA), social adversity (SA) and functional somatic symptoms (FSS) at four points in time—respondents aged 16, 21, 30 and 42. (DOCX)

S2 Table Direct and indirect effects in the model (5000 samples requested), stratified by sex (n = 473 women, 514 men). The variables are socioeconomic conditions (SC), occupational class (OC), material adversity (MA), social adversity (SA) and functional somatic symptoms (FSS), at four points in time—respondents aged 16, 21, 30 and 42.
(DOCX)
