# Supplementary material for: Life Course Pathways of Adversities Linking Adolescent Socioeconomic Circumstances and Functional Somatic Symptoms in Mid-Adulthood: A Path Analysis Study
Source: PLoS One. 2016 May 23;11(5):e0155963. doi: 10.1371/journal.pone.0155963 (PMC4877101; doi:10.1371/journal.pone.0155963)
Supplement: S1 Table — (DOCX) [file pone.0155963.s001.docx]

**S1 Table.** Pearson’s correlations between all the variables for women (above diagonal) and men (below diagonal); socioeconomic conditions (SC), occupational class (OC), material adversity (MA), social adversity (SA) and functional somatic symptoms (FSS) at four points in time – respondents aged 16, 21, 30 and 42.

| **Variables** | **SC16** | **OC21** | **OC30** | **MA21** | **MA30** | **SA21** | **SA30** | **FSS16** | **FSS42** |
| --- | --- | --- | --- | --- | --- | --- | --- | --- | --- |
| **SC16** | - | 0.163** | 0.134** | 0.095* | 0.225** | 0.056 | 0.149** | 0.021 | 0.027 |
| **OC21** | 0.291** | - | 0.407** | 0.101* | 0.186** | 0.028 | 0.158** | -0.003 | 0.022 |
| **OC30** | 0.266** | 0.473** | - | 0.154** | 0.304** | 0.017 | 0.228** | 0.003 | 0.083 |
| **MA21** | 0.019 | 0.133** | -0.018 | - | 0.309** | 0.069 | 0.130* | 0.007 | 0.042 |
| **MA30** | 0.168** | 0.224** | 0.241** | 0.131** | - | 0.129** | 0.272** | 0.059 | 0.175** |
| **SA21** | 0.022 | 0.002 | -0.006 | -0.006 | 0.115** | - | 0.113* | 0.086 | 0.106* |
| **SA30** | 0.104* | 0.140** | 0.218** | 0.051 | 0.237** | 0.023 | - | 0.133** | 0.175** |
| **FSS16** | 0.069 | 0.017 | 0.001 | -0.012 | 0.071 | 0.043 | -0.015 | - | 0.181** |
| **FSS42** | 0.123* | 0.139** | 0.152** | -0.007 | 0.178** | 0.025 | 0.222** | 0.253** | - |

*p < 0.05 (2-tailed), **p < 0.01 (2-tailed)
