# Supplementary material for: Life Course Pathways of Adversities Linking Adolescent Socioeconomic Circumstances and Functional Somatic Symptoms in Mid-Adulthood: A Path Analysis Study
Source: PLoS One. 2016 May 23;11(5):e0155963. doi: 10.1371/journal.pone.0155963 (PMC4877101; doi:10.1371/journal.pone.0155963)
Supplement: S2 Table — The variables are socioeconomic conditions (SC), occupational class (OC), material adversity (MA), social adversity (SA) and functional somatic symptoms (FSS), at four points in time—respondents aged 16, 21, 30 and 42. (DOCX) [file pone.0155963.s002.docx]

|  | **Women** | | | | **Men** | | | | |
| --- | --- | --- | --- | --- | --- | --- | --- | --- | --- |
| ***Total effect ^b^*** | **B (S.E.)** | **CI** | **β (S.E.)** | **CI** | **B (S.E.)** | **CI** | **β (S.E.)** | **CI** | |
| *SC16 🡪 FSS42* | 0.178 (0.351) | -0.510, 0.865 | 0.025 (0.048) | -0.070, 0.119 | 0.641 (0.273) | 0.106, 1.175 | 0.103 (0.043) | 0.019, 0.188 | |
| ***Direct effects ^a^*** | **Estimate** | | **CI** | | **Estimate** | | **CI** | | |
| *SC16 🡪 FSS42* | 0.089 (0.342) | | -0.639, 0.816 | | 0.167 (0.307) | | -0.473, 0.806 | | |
| *SC16 🡪 OC21 (probit)* | 0.560 (0.121) | | 0.308, 0.811 | | 0.949 (0.126) | | 0.705, 1.193 | | |
| *SC16 🡪 MA21* | 0.211 (0.067) | | 0.071, 0.352 | | 0.029 (0.061) | | -0.093, 0.152 | | |
| *SC16 🡪 SA21* | 0.123 (0.083) | | -0.048, 0.293 | | 0.028 (0.074) | | -0.109, 0.166 | | |
| *OC21 🡪 OC30 (probit)* | 0.781 (0.108) | | 0.560, 1.001 | | 0.836 (0.112) | | 0.609, 1.062 | | |
| *OC21 🡪 MA30* | 0.201 (0.053) | | 0.080, 0.322 | | 0.275 (0.044) | | 0.179, 0.371 | | |
| *OC21 🡪 SA30* | 0.186 (0.054) | | 0.070, 0.303 | | 0.182 (0.049) | | 0.078, 0.287 | | |
| *OC21 🡪 FSS42* | -0.221 (0.292) | | -0.865, 0.423 | | 0.214 (0.303) | | -0.412, 0.839 | | |
| *MA21 🡪MA30* | 0.436 (0.065) | | 0.267, 0.605 | | 0.232 (0.054) | | 0.114, 0.350 | | |
| *MA21 🡪SA30* | 0.125 (0.052) | | -0.035, 0.285 | | 0.102 (0.059) | | -0.041, 0.245 | | |
| *SA21 🡪 SA30* | 0.101 (0.047) | | -0.004, 0.207 | | 0.018 (0.055) | | -0.086, 0.123 | | |
| *OC30 🡪 FSS42* | 0.149 (0.231) | | -0.349, 0.647 | | 0.144 (0.206) | | -0.306, 0.594 | | |
| *MA30 🡪 FSS42* | 0.426 (0.157) | | 0.013, 0.840 | | 0.234 (0.143) | | -0.160, 0.627 | | |
| *SA30 🡪FSS42* | 0.421 (0.130) | | 0.022, 0.819 | | 0.536 (0.127) | | 0.235, 0.836 | | |
| **Total indirect effects ^b^** | **B (S.E.)** | **CI** | **β (S.E.)** | **CI** | **B (S.E.)** | **CI** | **β (S.E.)** | | **CI** |
| *SC16 🡪 FSS42* | 0.089 (0.134) | -0.174, 0.352 | 0.012 (0.018) | -0.024, 0.048 | 0.474 (0.179) | 0.123, 0.825 | 0.076 (0.028) | | 0.021, 0.132 |
| ***Specific indirect ^b^*** |  |  |  |  |  |  |  | |  |
| *SC16 🡪 OC21 🡪 OC30 🡪 FSS42* | 0.065 (0.119) | -0.167, 0.298 | 0.009 (0.016) | -0.023, 0.041 | 0.114 (0.191) | -0.260, 0.489 | 0.018 (0.031) | | -0.042, 0.079 |
| *SC16 🡪 OC21 🡪 MA30 🡪 FSS42* | 0.048 (0.035) | -0.020, 0.116 | 0.007 (0.005) | -0.003, 0.016 | 0.061 (0.054) | -0.045, 0.166 | 0.010 (0.009) | | -0.007, 0.027 |
| *SC16 🡪 OC21 🡪 SA30 🡪 FSS42* | 0.044 (0.032) | -0.019, 0.106 | 0.006 (0.004) | -0.003, 0.015 | 0.093 (0.038) | 0.019, 0.166 | 0.015 (0.006) | | 0.003, 0.027 |
| *SC16 🡪 MA21 🡪 MA30 🡪 FSS42* | 0.039 (0.030) | -0.019, 0.097 | 0.005 (0.004) | -0.002, 0.013 | 0.002 (0.005) | -0.008, 0.011 | 0.000 (0.001) | | -0.001, 0.002 |
| *SC16 🡪 MA21 🡪 SA30 🡪 FSS42* | 0.011 (0.013) | -0.015, 0.037 | 0.002 (0.002) | -0.002, 0.005 | 0.002 (0.005) | -0.007, 0.011 | 0.000 (0.001) | | -0.001, 0.002 |
| *SC16 🡪 SA21 🡪 SA30 🡪 FSS42* | 0.005 (0.007) | -0.008, 0.019 | 0.001 (0.001) | -0.001, 0.003 | 0.000 (0.002) | -0.005, 0.005 | 0.000 (0.000) | | -0.001, 0.001 |

**S2 Table.** Direct and indirect effects in the model (5000 samples requested), stratified by sex (n = 473 women, 514 men). The variables are socioeconomic conditions (SC), occupational class (OC), material adversity (MA), social adversity (SA) and functional somatic symptoms (FSS), at four points in time – respondents aged 16, 21, 30 and 42.

^a^ Predictor estimates for the direct effects are unstandardized path coefficients (S.E.) and 95 % confidence intervals (CI ). ^b^ Predictor estimates for indirect, and specific indirect effects are unstandardized path coefficients (B) with bootstrapped standard errors (S.E.) and bootstrapped 95 % confidence intervals (CI) and standardized path coefficients (β) with bootstrapped standard errors (S.E.) and bootstrapped 95 % confidence intervals (CI).
